# Abrogation of CC chemokine receptor 9 ameliorates ventricular remodeling in mice after myocardial infarction

**DOI:** 10.1038/srep32660

**Published:** 2016-09-02

**Authors:** Yan Huang, Dandan Wang, Xin Wang, Yijie Zhang, Tao Liu, Yuting Chen, Yanhong Tang, Teng Wang, Dan Hu, Congxin Huang

**Affiliations:** 1 Department of Cardiology, Renmin Hospital of Wuhan University, Wuhan 430060, PR China; 2 Cardiovascular Research Institute, Wuhan University, Wuhan 430060, PR China; 3 Hubei Key Laboratory of Cardiology, Wuhan 430060, PR China; 4 Masonic Medical Research Laboratory, Utica, NY, USA

## Abstract

CC chemokine receptor 9 (CCR9), which is a unique receptor for CC chemokine ligand (CCL25), is mainly expressed on lymphocytes, dendritic cells (DCs) and monocytes/macrophages. CCR9 mediates the chemotaxis of inflammatory cells and participates in the pathological progression of inflammatory diseases. However, the role of CCR9 in the pathological process of myocardial infarction (MI) remains unexplored; inflammation plays a key role in this process. Here, we used CCR9 knockout mice to determine the functional significance of CCR9 in regulating post-MI cardiac remodeling and its underlying mechanism. MI was induced by surgical ligation of the left anterior descending coronary artery in CCR9 knockout mice and their CCR9+/+ littermates. Our results showed that the CCR9 expression levels were up-regulated in the hearts of the MI mice. Abrogation of CCR9 improved the post-MI survival rate and left ventricular (LV) dysfunction and decreased the infarct size. In addition, the CCR9 knockout mice exhibited attenuated inflammation, apoptosis, structural and electrical remodeling compared with the CCR9+/+ MI mice. Mechanistically, CCR9 mainly regulated the pathological response by interfering with the NF-κB and MAPK signaling pathways. In conclusion, the data reveal that CCR9 serves as a novel modulator of pathological progression following MI through NF-κB and MAPK signaling.

Myocardial infarction (MI), which results from ischemic damage to the left ventricle (LV), is a leading cause of morbidity and mortality in humans worldwide^1^. MI triggers an intense pathological process that includes inflammation, apoptosis, cardiomyocyte hypertrophy, and fibrosis and can lead to post-infarction remodeling, heart failure, arrhythmias and, thus, sudden cardiac death^2^. There have been some major accomplishments in MI therapy, including electrophysiological monitoring, the routine use of beta blockers, aspirin and other medications, and reperfusion therapy. Although these improvements have reduced the acute mortality of MI^3^,^4^, patients surviving a large MI are likely to develop heart failure, and the incidence of heart failure following MI continues to increase. Therefore, it is of great importance to reveal the mechanisms underlying cardiac remodeling that involve complicated interactions among cardiomyocytes, inflammatory cells and fibroblasts and to explore new measures that can prevent the progression of the pathological process.

Chemokines, which include four subfamilies, C, CC, CXC, and CX3C, are a family of small, highly basic proteins with low molecular weight that ranges from 8 to 14 kDa^5^,^6^. Chemokines play a critical role in leukocyte trafficking in both homeostasis and inflammation by interacting with the already known chemokine receptors (CCR1-10)^7^. Chemokine receptors are seven transmembrane-spanning heterotrimeric G protein-coupled receptors that are mainly expressed on both immune cells and other nonimmune cells, such as tumor cells and endothelial cells^8^. Chemokines and chemokine receptors interact with each other to stimulate the migration of lymphocytes, monocytes, neutrophils, endothelial cells and mesenchymal stem cells as well as tumor progression, angiogenesis and metastasis^9^.

Similar to other chemokine receptors, CC chemokine receptor 9 (CCR9) is a G-protein coupled receptor with only one known ligand, thymus-expressed chemokine/CCL25^10^. CCR9 is preferentially expressed on lymphocytes, dendritic cells (DCs) and monocytes/macrophages^11^. Originally, CCR9 was found to be involved in the migration and development of T cells in the thymus^12^. However, in recent years, emerging evidence has shown that CCR9 participates in several diseases^13^,^14^,^15^,^16^,^17^,^18^. Some studies have been conducted to interfere with the CCR9/CCL25 axis and test its effectiveness. The use of neutralizing antibodies in animal disease models has proven to be effective^14^,^16^,^19^,^20^,^21^,^22^. CCR9−/− mice did not develop hepatitis following conA injection^14^, and the abrogation of CCR9 ameliorates collagen-induced arthritis in mice^23^. However, other studies have shown that CCR9−/− mice are more susceptible to DSS-induced colitis^22^,^24^. Myocardial infarction is associated with inflammatory responses that involve a variety of chemokines^6^. However, it is unknown whether CCR9 plays a role in the post-MI pathological processes.

In this study, we used CCR9 knockout mice to determine the role of CCR9 in the mouse heart after MI. The results indicated that cardiac remodeling after MI was blunted in the CCR9-deficient mice. Moreover, we discovered that these beneficial outcomes were somehow dependent on the NF-κB and mitogen-activated protein kinase (MAPK) signaling pathways.

## Results

### CCL25 and CCR9 were up-regulated in mouse infarcted hearts

**Figure 1:**
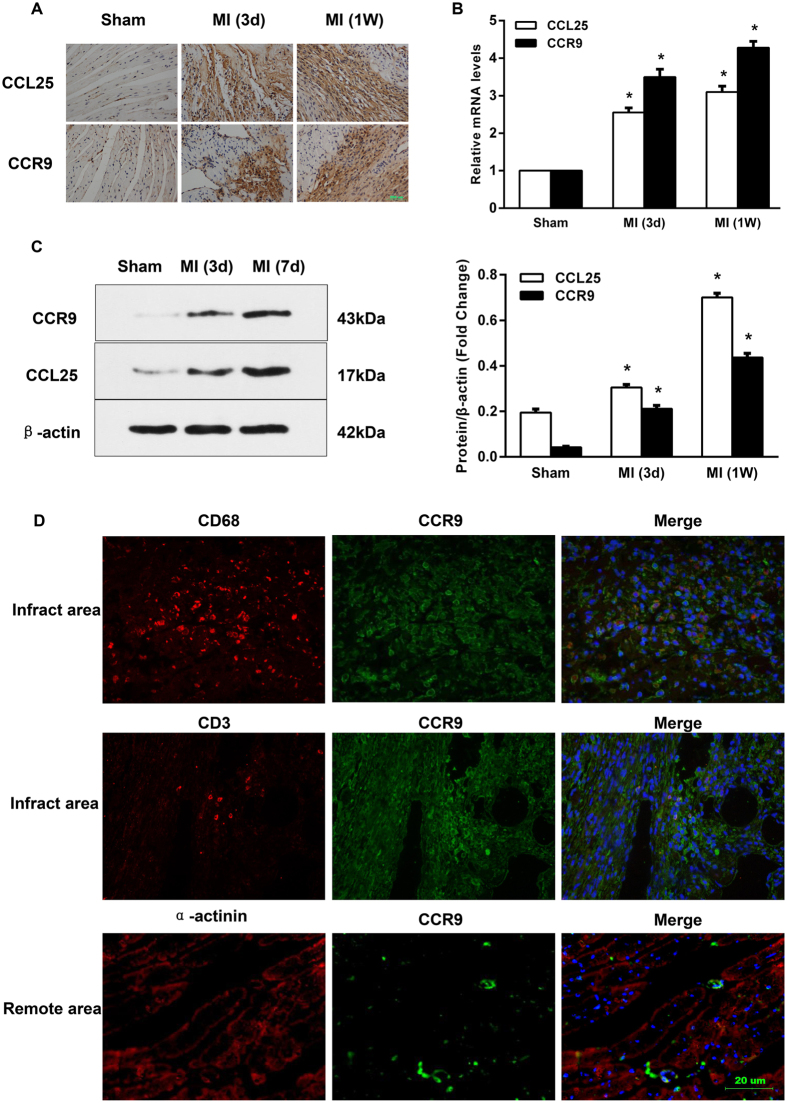
CCR9 and CCL25 expression in wild-type mouse hearts following MI. (**A**) CCR9 and CCL25 expression in the infarct zone and border zone. Immunohistochemical staining with a rat anti-mouse CCL25 mAb or goat anti-mouse CCR9 was performed on heart sections from wild-type mice 3 days or 1 week after the sham operation or MI surgery (n = 4). (**B**) Levels of the CCR9 and CCL25 mRNAs in heart tissues from wild-type mice 3 days or 1 week after the sham operation or MI surgery (n = 4, *P < 0.05 versus their littermate shams). (**C**) Representative western blots of the CCR9 and CCL25 protein levels in wild-type mouse heart tissues at 3 days or 1 week after the sham operation or MI surgery (n = 4, *P < 0.05 versus their littermate shams). The original bands are presented in Supplementary Figure S3. (**D**) Representative immunofluorescent colocalization for CCR9 (green), CD68 (red)/CD3 (red)/a-actinin (red), and the nuclei (blue) on heart sections from wild-type mice 1 week after the MI surgery (n = 4).

To investigate the role of CCR9 in heart pathology after MI, we first examined the expression levels of CCR9 and its ligand CCL25 in CCR9+/+ mouse hearts following MI and a sham operation. The immunohistochemistry results showed that the expression of both CCL25 and CCR9 was significantly up-regulated at 3 and 7 days after MI, whereas the sham hearts displayed low expression levels (Fig. 1A). Immunohistochemistry also indicated that CCL25 and CCR9 were mainly expressed in the infarct area and border zone, in which a large number of inflammatory cells had infiltrated, revealing that CCR9 was highly expressed in infiltrating cells. Double immunofluorescence experiments were performed using an anti-CCR9 antibody combined with anti-CD68, anti-CD3 or anti-(α-actinin) antibodies to determine which cells expressed CCR9. The results showed that CCR9 was highly expressed in CD68-positive and CD3- positive cells but not cardiomyocytes (Fig. 1D). RT-PCR and western blots indicated the same results as immunohistochemistry. Both the CCR9 RNA and protein levels were up-regulated at 3 days post-MI, and the expression levels were even higher at 1 week after MI (Fig. 1B,C).

### CCR9 deletion reduced MI-induced mortality and infarct size and improved cardiac dysfunction

**Figure 2:**
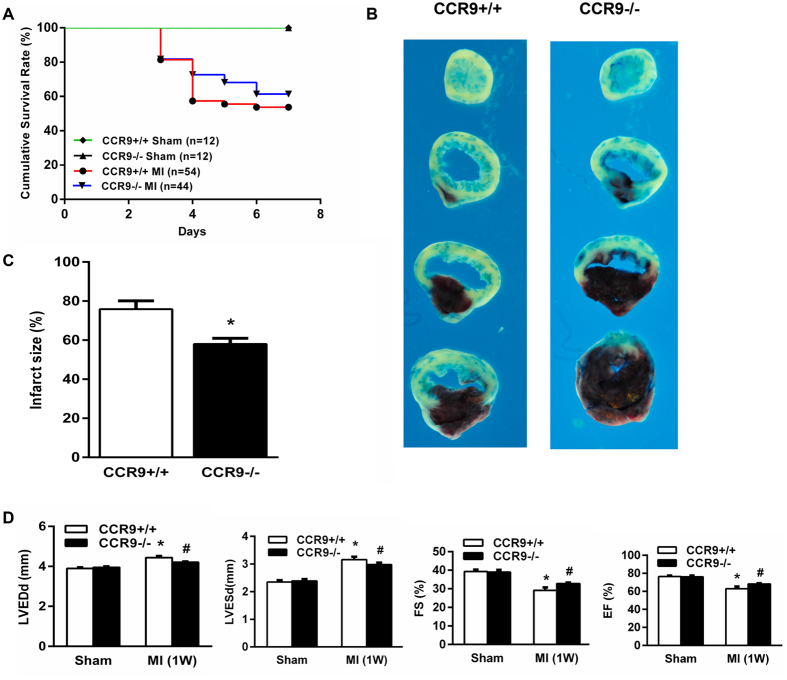
Deletion of CCR9 reduced mortality and the infarct size and improved cardiac function following MI. (**A**) Kaplan-Meier survival curves of the CCR9+/+ and CCR9−/− mice 1 week after the sham operation or MI surgery. (**B**) Representative Evans blue and TTC staining of CCR9+/+ and CCR9−/− mouse heart sections 24 h after the MI surgery. (**C**) Statistical analysis of the infarct size (%) in CCR9+/+ and CCR9−/− mice hearts 24 h after MI (n = 5, *P < 0.05 vs. CCR9+/+/MI). (**D**) Echocardiographic results of the CCR9+/+ and CCR9−/− mice 1 week after the sham operation or MI surgery (n = 8–10, *P < 0.05 vs. their littermate shams, ^#^P < 0.05 vs. CCR9+/+/MI).

The increased expression levels in the mouse heart after MI suggested that CCR9 may be involved in the pathological process after MI. CCR9 knockout mice were used, MI surgery or a sham operation was conducted, and the mice were observed for 1 week to clarify the role of CCR9. All sham-operated mice survived for 1 week. The CCR9−/− MI mice displayed a trend of increased (but not significant) survival rates (61.4%) compared with the CCR9+/+ MI mice (53.7%) at 1 week after MI (p > 0.05; Fig. 2A). Consistent with the survival rates, Evans blue dye and TTC staining showed that the CCR9−/− MI mice had a smaller infarct size than the CCR9+/+ MI mice (58.0% vs 75.9%, p < 0.05; Fig. 2B,C). The echocardiographic measurements showed improved cardiac function with a decreased LVEDd and LVESd and a higher FS and EF in the CCR9−/− MI group compared with the CCR9+/+ MI group (Fig. 2D).

### CCR9 deletion attenuated MI-induced cardiomyocyte death

**Figure 3:**
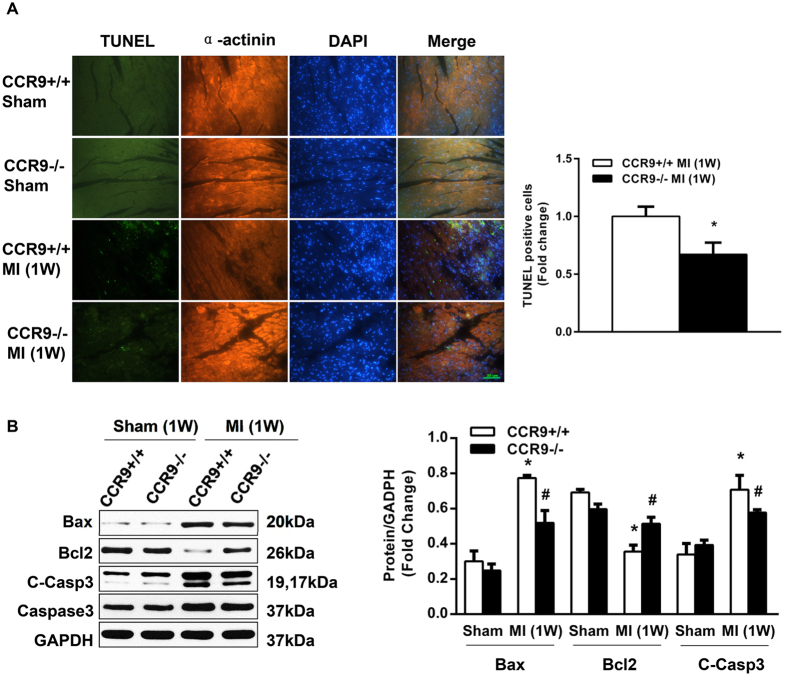
Loss of the CCR9 gene prevented cardiomyocyte apoptosis and regulated the expression of apoptosis-related genes following MI. (**A**) Representative photograph of immunofluorescent staining for TUNEL (green), a-actinin (red), and the nuclei (blue). Statistical analysis of TUNEL and a-actinin double-positive cells in the border area of CCR9+/+ and CCR9−/− mice hearts 1 week after the sham operation or MI surgery (n = 4, *P < 0.05 vs. CCR9+/+/MI). (**B**) Representative western blots and statistical analysis of the apoptosis-related proteins levels in CCR9+/+ and CCR9−/− mouse heart tissues 1 week after the sham operation or MI surgery (n = 4, *P < 0.05 vs. CCR9+/+/sham, ^#^P < 0.05 vs. CCR9+/+/MI). The original bands are presented in Supplementary Figure S4.

Oxidative stress, ischemia, and hypoxic injury and reperfusion can cause cardiomyocyte apoptosis following MI. TUNEL assays were used to determine the extent of cardiomyocyte apoptosis in the peri-infarcted area at 1 week after MI. A decreased number of TUNEL-positive cardiomyocytes was observed in the CCR9−/− MI hearts compared with the CCR9+/+ MI hearts (Fig. 3A). Further, we examined the changes in the expression of the pro-apoptotic proteins Bax and cleaved caspase 3 and the anti-apoptotic protein Bcl2. Western blots indicated that Bcl2 expression was markedly increased in the CCR9−/− MI hearts, whereas the expression of Bax and cleaved caspase 3 was decreased compared with the CCR9+/+ MI hearts (Fig. 3B).

### CCR9 deficiency inhibited inflammatory responses following MI

**Figure 4:**
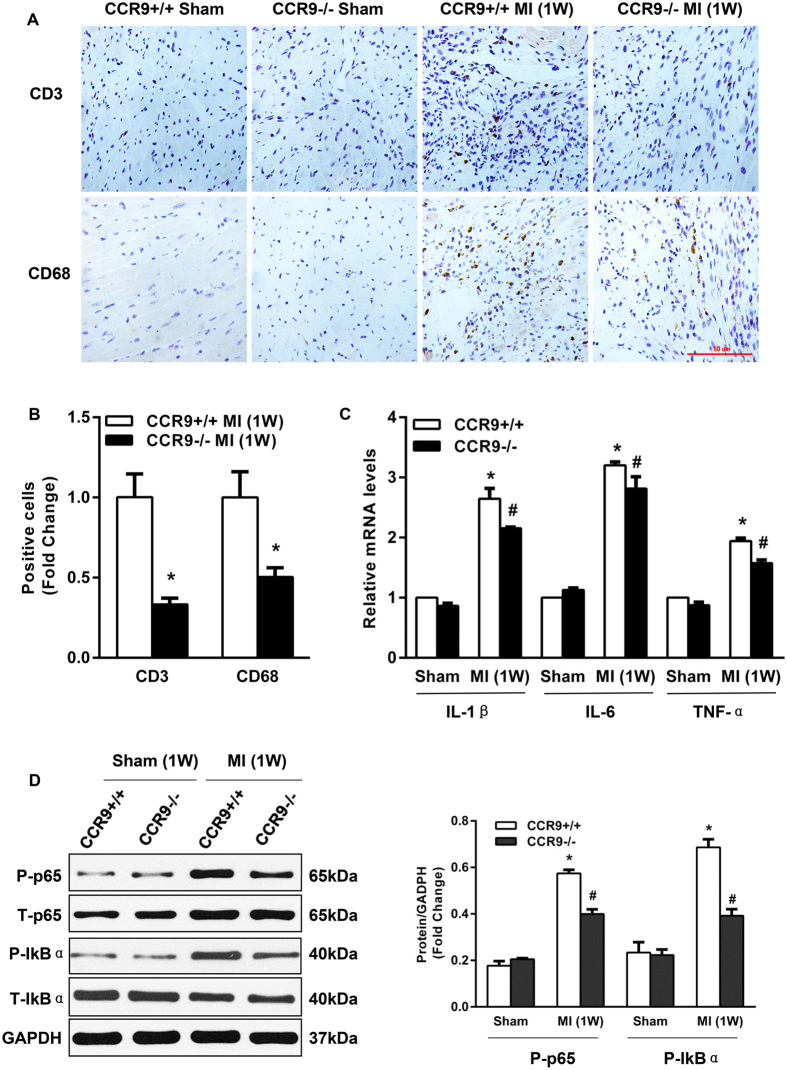
Abrogation of CCR9 attenuated inflammation in response to MI. (**A**) Immunohistochemical staining for the CD3-positive and CD68-positive cells in heart sections from the CCR9+/+ and CCR9−/− mice 1 week after the sham operation or MI surgery (n = 4). (**B**) Statistical analysis of the CD3-positive cells and CD68-positive cells in CCR9+/+ and CCR9−/− mouse hearts 1 week after the sham operation or MI surgery (n = 4, *P < 0.05 vs. CCR9+/+/MI). (**C**) Levels of proinflammatory cytokine mRNAs in the CCR9+/+ and CCR9−/− mouse heart tissues 1 week after the sham operation or MI surgery (n = 4, *P < 0.05 vs. CCR9+/+/sham, ^#^P < 0.05 vs. CCR9+/+/MI). (**D**) Representative western blots and statistical analysis of p65 and IκBa in CCR9+/+ and CCR9−/− mouse heart tissues 1 week after the sham operation or MI surgery (n = 4, *P < 0.05 vs. CCR9+/+/sham, ^#^P < 0.05 vs. CCR9+/+/MI). The original bands are presented in Supplementary Figure S5.

Inflammation is the most important pathological response for damage and repair. Moreover, CCR9 mainly acts on the chemotaxis of inflammatory cells. The inflammatory phase starts with the rapid infiltration of neutrophils. From day 3 to day 7, the inflammatory cells are mainly monocyte-derived macrophages and T-lymphocytes. Therefore, we took advantage of immunohistochemistry to detect monocyte-derived macrophages and T-lymphocytes in the infarcted zone at 1 week after MI. The immunohistochemistry analysis presented a decreased infiltration of these inflammatory cells in the CCR9−/− MI hearts at 1 week after MI (Fig. 4A,B). We also explored the expression levels of cytokines secreted by inflammatory cells. The mRNA levels of the proinflammatory cytokines IL-1β, IL-6 and TNF-α were decreased in the CCR9−/− MI LV tissue (Fig. 4C). Considering that the NF-κB pathway largely mediates the inflammatory response, we next studied the expression levels of proteins involved in the NF-κB pathway. As shown in Fig. 4D, the levels of phosphorylated p65 and IκBα were substantially reduced in the knockout MI mice, indicating that the NF-κB pathway was strongly suppressed by the CCR9 deletion.

### CCR9 deletion attenuated LV remodeling by influencing MAPK signaling pathway

**Figure 5:**
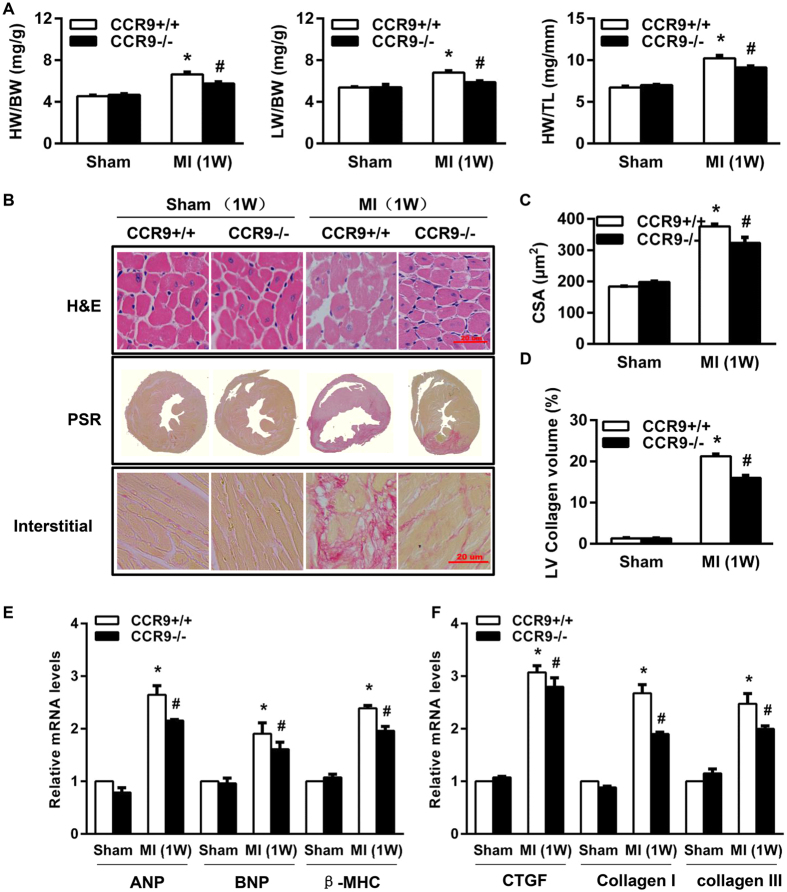
Knockout of the CCR9 gene improved cardiac structural remodeling after MI. (**A**) HW/BW, LW/BW, and HW/TL ratios in CCR9+/+ and CCR9−/− mice 1 week after the sham operation or MI surgery (n = 11–16, *P < 0.05 vs. CCR9+/+/sham, ^#^P < 0.05 vs. CCR9+/+/MI). (**B**) Hematoxylin and eosin (H&E) staining and PSR staining of CCR9+/+ and CCR9−/− mouse hearts 1 week after the sham operation or MI surgery (n = 4). (**C**) Statistical analysis of the cardiomyocyte cross-sectional area (CSA) from H&E-stained slices of CCR9+/+ and CCR9−/− mouse hearts 1 week after the sham operation or MI surgery (n = 100+ cardiomyocytes in four samples, *P < 0.05 vs. CCR9+/+/sham, ^#^P < 0.05 vs. CCR9+/+/MI). (**D**) Statistical analysis of the LV collagen volume (%) in PSR-stained slices of CCR9+/+ and CCR9−/− mouse hearts 1 week after the sham operation or MI surgery (n  =  25+ fields in four samples, *P < 0.05 vs. CCR9+/+/sham, ^#^P < 0.05 vs. CCR9+/+/MI). (**E**) Levels of hypertrophy-related mRNAs in CCR9+/+ and CCR9−/− mouse heart tissues 1 week after the sham operation or MI surgery (n = 4, *P < 0.05 vs. CCR9+/+/sham, ^#^P < 0.05 vs. CCR9+/+/MI). F) Levels of fibrosis-related mRNAs in CCR9+/+ and CCR9−/− mouse heart tissues 1 week after the sham operation or MI surgery (n = 4, *P < 0.05 vs. CCR9+/+/sham, ^#^P < 0.05 vs. CCR9+/+/MI).

**Figure 6:**
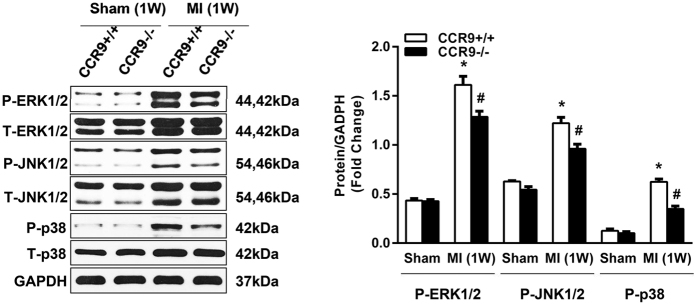
The loss of CCR9 improved cardiac remodeling by regulating the MAPK signaling pathway. Representative western blots and statistical analysis of ERK1/2, JNK1/2 and p38 in CCR9+/+ and CCR9−/− mouse heart tissues 1 week after the sham operation or MI surgery (n = 4, *P < 0.05 vs. CCR9+/+/sham, ^#^P < 0.05 vs. CCR9+/+/MI). The original bands are presented in Supplementary Figure S6.

Structural remodeling, including hypertrophy and interstitial fibrosis, can ultimately result in heart failure. Thus, we further investigated whether the CCR9 gene had an effect on cardiac remodeling following MI and the extent of its effects. At 1 week post-MI, the CCR9−/− MI mice exhibited lower heart weight (HW)/body weight (BW), lung weight (LW)/body weight and heart weight/tibia length (TL) ratios (Fig. 5A). The cross-sectional area (CSA) of cardiomyocytes in a remote area, as analyzed by H&E staining, was much smaller in the CCR9−/− MI mice than in the CCR9+/+ MI mice; the morphologies of the cardiomyocytes were more regular and the LV collagen volume was much smaller in the CCR9−/− MI mice (Fig. 5B–D). In addition, the mRNA levels for markers of hypertrophy (ANP, BNP and β-MHC) and fibrosis (CTGF, collagen I, and collagen III) were all reduced in the CCR9−/− mice at 1 week post-MI (Fig. 5E,F). Because the MAPK (mitogen-activated protein kinase) signaling pathway plays a vital role in cardiac hypertrophy, we further examined the levels of ERK1/2, JNK1/2 and p38 expression and phosphorylation. After MI, the levels of phosphorylated ERK1/2, JNK1/2 and p38 were all remarkably increased, but they were reduced in the CCR9−/− MI mice (Fig. 6). We also tried to monitor the pathways (PI3K/AKT and Smad) associated with fibrosis, but we did not observe any difference between the CCR9+/+ MI mice and the CCR9−/− MI mice (Supplementary Figure S2). These data hinted that the CCR9 deletion inhibited cardiac remodeling by affecting the MAPK pathway to some extent.

### CCR9 knockdown mice had similar telemetry electrocardiogram (ECG) changes as did CCR9+/+ mice after MI

**Figure 7:**
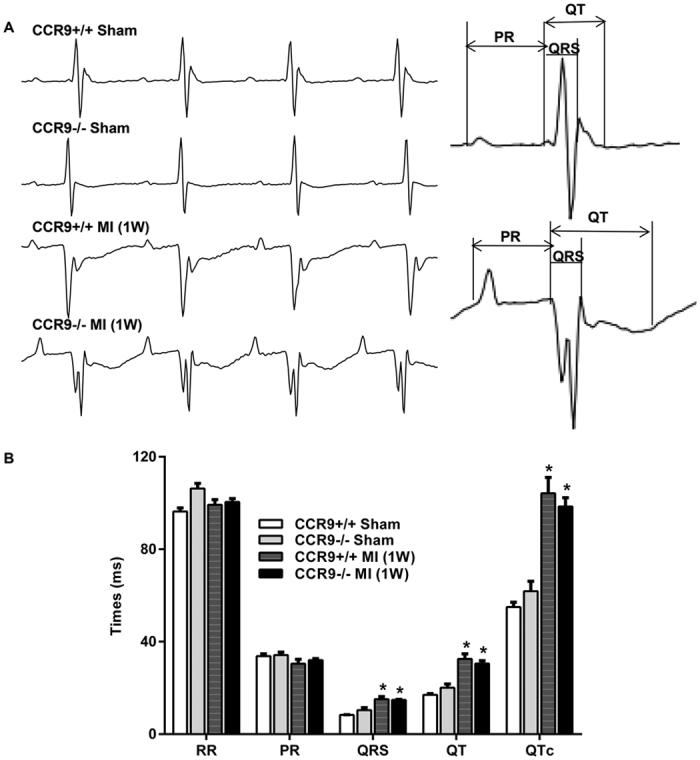
CCR9 knockout did not change the ECG parameters after MI. (**A**) Representative ECG recordings in CCR9+/+ and CCR9−/− mice 1 week after the sham operation or MI surgery (n = 6–8). (**B**) Statistical analysis of the RR, PR, QRS, QT and QTc intervals in CCR9+/+ and CCR9−/− mouse telemetries 1 week after the sham operation or MI surgery (n = 6–8, *P < 0.05 vs. their littermate shams).

Because we observed improved structural remodeling in the CCR9−/− mice after MI, we further contemplated that silencing of the CCR9 gene would have an influence on the cardiac electrophysiological properties. Therefore, we first monitored the electric signals using implantable telemetry. During 24 h of continuous recording, the heart rate, RR interval, and PR interval were approximately the same among the four groups. Both the CCR9+/+ and CCR9−/− mice had a prolonged QRS interval, QT interval and QTc (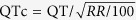 ) after MI. However, there was no difference between the CCR9+/+ MI mice and CCR9−/− MI mice (Fig. 7A,B).

### CCR9 deficiency reduced 90% action potential duration (APD90) and increased threshold of action potential duration alternans and susceptibility to ventricular arrhythmia (VA) following MI

**Figure 8:**
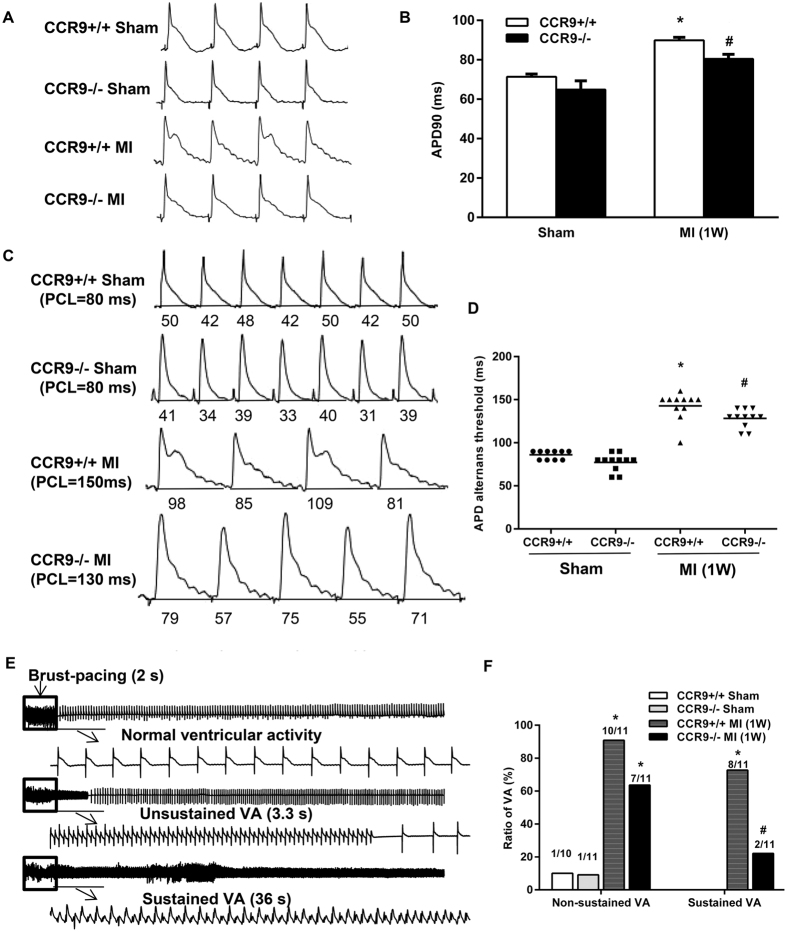
The CCR9 deletion improved electrophysiological properties of the heart *in vitro*. (**A,B**) Representative action potential figures and statistical analysis of the 90% action potential durations in CCR9+/+ and CCR9−/− mouse hearts 1 week after the sham operation or MI surgery (n = 10–11, *P < 0.05 vs. CCR9+/+/shams, ^#^P < 0.05 vs. CCR9+/+/MI). (**C,D**) Representative electric alternans figures and statistical analysis of the ALT thresholds in CCR9+/+ and CCR9−/− mouse hearts 1 week after the sham operation or MI surgery (n = 10–11, *P < 0.05 vs. CCR9+/+/shams, ^#^P < 0.05 vs. CCR9+/+/MI). (**E,F**) Representative arrhythmia induced by burst-pacing stimulations and statistical analysis of CCR9+/+ and CCR9−/− mouse hearts 1 week after the sham operation or MI surgery (n = 10–11, *P < 0.05 vs. their littermate shams, ^#^P < 0.05 vs. CCR9+/+/MI).

Langendorff-perfused hearts were used to characterize the changes in the electrophysiological parameters (APD90, threshold of APD alternans and VA incidence). APD90 was measured under the conditions in which the Langendorff-perfused hearts were administered a programmed electrical stimulation with a pacing cycle length of 150 ms. After MI, the APD90 was significantly prolonged, but it became much shorter in the CCR9−/− MI mice compared with the CCR9+/+ MI mice (Fig. 8A,B). The threshold of APD alternans was significantly decreased after MI but was increased in the CCR9 knockout mice (Fig. 8C,D). Additionally, non-sustained ventricular arrhythmias were induced in 10 of the 11 CCR9+/+ MI mice, and 7 CCR9−/− MI mice incurred non-sustained ventricular arrhythmias, but there was no significant difference (p > 0.05, Fig. 8E,F). The ratio of sustained ventricular arrhythmias was reduced in the CCR9−/− MI mice compared with the CCR9+/+ MI mice (Fig. 8E,F).

## Discussion

In the present study, we originally showed that the abrogation of CCR9 improved cardiac structural remodeling and electrical remodeling in a murine model of MI. The main findings of this study are listed below. (1) CCR9 expression was markedly up-regulated in mouse hearts after MI. (2) CCR9 knockdown could reduce post-MI mortality, infarct size and improve cardiac function, which may result from alleviated inflammation, reduced cardiomyocyte apoptosis and mitigated fibrosis. (3) CCR9 was mainly involved in structural remodeling by interfering with the NF-κB and MAPK signaling pathways. (4) The CCR9 knockout had beneficial effects on electrical remodeling because of the shorter APD90 and the increased threshold of APD alternans. These findings suggest that the CCR9-CCL25 axis may play an important role in inflammatory cell infiltration and cardiac remodeling after MI.

Emerging evidence has shown that chemokines play a critical role in early myocardial dysfunction and disease progression following MI by regulating infarct angiogenesis, leukocyte infiltration and fibrous tissue deposition^25^,^26^,^27^. The infarct size can be reduced and cardiomyocyte injury can be prevented by targeting chemokine-mediated inflammation in the post-MI heart^28^,^29^,^30^,^31^,^32^,^33^. CCR9, a chemokine receptor that mediates the chemotaxis of inflammatory cells in combination with CCL25 (a member of CC chemokine superfamily), was identified and has recently received recognition^10^,^34^. CCR9 has been documented in various tissues, such as the small intestine, liver, arthritic tissues, and the brain, as mainly mediating inflammatory diseases^13^,^14^,^15^,^16^,^35^. In the cardiovascular system, CCR9 was first found to be associated with the pathophysiology of atherosclerosis, and inhibition of CCR9 by RNA interference in apoE-deficient mice significantly retarded the development of atherosclerosis^18^. Recently, Xu Z *et al*. reported that CCR9 had a causal role in pathological cardiac hypertrophy. They found that overexpression of CCR9 exacerbated pressure overload–induced cardiac hypertrophy and dysfunction^36^. However, it has not been investigated whether CCR9 is involved in other cardiovascular diseases. Because MI is closely related to the inflammatory response, which is the inevitable result of atherosclerosis and associated with heart failure, we tried to explore its role and molecular mechanisms in the pathological process of MI. Consistent with our hypothesis, our findings suggested that CCR9 participated in and regulated cardiac structural remodeling and electrical remodeling following MI by regulating the NF-κB and MAPK signaling pathways.

Upon myocardial ischemic injury, dying cardiomyocytes release danger signals and pathogenic assaults to activate immune pathways by inducing chemokine and cytokine synthesis and recruiting neutrophils, mononuclear cells and lymphocytes^37^. These danger signals, including inflammatory cytokines and stress, were thought to evoke NF-κB signaling and promote the expression of pro-inflammatory genes^38^. Induced chemokines, such as CXC and CC chemokines, bind to the endothelial surface and attract neutrophils and mononuclear cells to the infarcted area. These leukocytes secrete cytokines and chemokines to mediate cardiac repair at the early stage, but excessive activation of inflammation has negative inotropic effects and proteases can degrade the matrix, leading to cardiac cell death and adverse remodeling in the MI heart^37^,^38^,^39^. Furthermore, these pro-inflammatory cytokines, such as TNF and IL-1, could also be a stimulus to activate NF-κB^38^,^40^, which we believe to be a vicious circle. In addition, inhibition of NF-κB or IκBα can improve survival and remodeling by decreasing cytokine expression and cardiomyocyte apoptosis^39^,^41^. In the present study, CCR9 deletion could diminish the activation of the NF-κB pathway and inhibit cytokine expression, which likely explained some of the anti-inflammatory and anti-apoptotic effects of CCR9 abrogation.

MI refers to a complicated pathological process that involves multiple signal pathways. The MAPK signaling pathway plays a vital role in post-infarct remodeling and heart failure^33^. Injury factors, including inflammatory cytokines, stress, and ischemia/reperfusion, lead to the activation of MAPKs^42^. The MAPK signal transduction pathway is linked to a series of cardiac pathologies, including inflammation, apoptosis, cardiac hypertrophy and heart failure^43^,^44^. NF-κB was reported to be a downstream component of the MAPK pathway^45^. Consistent with a previous study^46^, the phosphorylation of the ERK, JNK, and p38 MAPK subunits were ultimately increased in response to injuries after MI. ERK activation was thought to promote cell growth and migration; therefore, the effects of ERK1/2 activation may be complicated because it promoted fibroblast differentiation after MI but had cytoprotective effects on cultured cardiomyocytes following ischemia/oxygenation^47^,^48^. Whereas, p38 and JNK activation promote myocardial apoptosis, cardiac dysfunction and fibrosis^43^, inhibition of p38 MAPK decreased the infarct size, improved heart function and attenuated myocardial fibrosis^49^,^50^ and inhibition of JNK suppressed LPS-induced cardiac myocyte apoptosis^51^. In the present study, ERK, JNK and p38 were all inhibited in response to CCR9 deletion. Therefore, we deduced that the MAPK pathway may be a vital underlying mechanism by which CCR9 regulated cardiac remodeling. However, in the report of Xu Z *et al*.^36^, they found AKT signaling might be the underlying mechanism of prohypertrophic effects of CCR9, which was different from our findings. We thought this was mainly because the different animal models.

The surviving cardiomyocytes in the peri-infarction area experience not only structural remodeling but also dramatic electrical remodeling, which is essential to trigger ventricular arrhythmia^52^. Susceptibility to arrhythmia may be derived from the prolonged action potential duration and decreased ALT threshold in the infarct border zone^53^,^54^. The molecular mechanisms responsible for these electrophysiological changes are still unclear. However, it is important that key cytokines and chemokines, such as IL-1β, IL-6, TNF-α, and MMPs, that are elevated in the heart tissue following MI cause electrophysiological changes in the peri-infarction zone, suggesting that inflammation may be a critical mediator of electrophysiological remodeling and arrhythmia^52^. Indeed, various clinical and pre-clinical studies have shown that elevated inflammatory factors were related to a remarkably increased risk for ventricular arrhythmias^55^,^56^,^57^. Thus, we believe that the inhibition of inflammation, as well as cytokines and chemokines, will make a difference in preventing the development of arrhythmia.

In conclusion, for the first time, this study shows that the loss of the CCR9 gene protected the mice from MI-induced inflammation, apoptosis, hypertrophy, fibrosis, and arrhythmia by regulating the NF-κB and MAPK pathways. Therefore, these findings suggest that a promising strategy for preventing or treating structural remodeling and electrical remodeling following MI may be to target the CCR9-CCL25 axis. Although, we have some new findings, we have some limitations, too. We didn’t conduct experiments on cell levels, Xu Z *et al*.^36^ showed that CCR9 was expressed on the neonatal rat cardiomyocytes, but we didn’t observe obvious expression on cardiomyocytes from mouse heart sections. Therefore, we plan to develop further research on cell levels.

## Methods

### Experimental animals

Global CCR9 knockout mice (CCR9-KO, C57BL/6J background; Supplementary Table S3 and Figure S1) were purchased from the European Mouse Mutant Archive (EM:02293. B6;129-Ccr9tm1Dgen/H). The mice were housed and bred in our specific pathogen-free (SPF) facility. All mice used in this study were males, with body weights ranging from 24 to 27 g. All animal experiments were performed in compliance with the National Institute of Health Guide for the Care and Use of Laboratory Animals (NIH Publication No. 85–23, revised 1996). Moreover, all experimental protocols were approved by the Animal Care and Use Committee of Renmin Hospital of Wuhan University.

### Left coronary artery ligation

Mice were anesthetized with an intraperitoneal injection of 50 mg/kg sodium pentobarbital. After full anesthesia was achieved, artificial respiration was performed to maintain a normal pH, PO_2_ and PCO_2_. In the experimental groups, the pericardium was opened with a thoracotomy at the third or fourth intercostal space, and the left anterior descending coronary was ligated using a 7–0 silk suture. The sham-operated animals underwent the same surgical procedure without ligation^58^.

### Echocardiography assessment of heart function

Echocardiography was performed under light anesthesia with 1.5–2% isoflurane to examine the left ventricle function of the mice using the MyLab 30CV system (Biosound Esaote, Inc.). Standard 2D echocardiographic images of the left ventricular (LV) dimensions were collected in the short-axis view at the papillary muscle level. M-mode measurement data were obtained from an average of 4–5 cardiac cycles. The LV end-diastolic dimension (LVEDd), LV end-systolic diameter (LVESd), ejection fraction (EF) and LV fractional shortening (FS) were measured^59^.

### Histology, immunohistochemistry, and immunofluorescence

The mice were sacrificed 3 days or 1 week after MI. The hearts were removed and rinsed with phosphate-buffered saline (PBS). The hearts were arrested in diastole with 10% KCl, then fixed in 10% buffered formalin for 24 h, and embedded in paraffin. Three-micrometer-thick sections were cut and stained with hematoxylin and eosin (H&E) for the morphometric analysis or with picrosirius red (PSR) to examine interstitial collagen deposition. For immunohistochemistry, the sections were stained with a rat anti-mouse CCL25 monoclonal antibody (sc80344; Santa, Texas, USA) or a goat anti-mouse CCR9 antibody (ab1662; Abcam, Cambridge, UK) to examine the expression of CCL25 and CCR9. For the cluster of differentiation proteins, a CD3 antibody (sc20047; Santa, Texas, USA) and CD68 antibody (sc20060; Santa, Texas, USA) were used to examine T-lymphocyte and macrophage infiltration, respectively. For immunofluorescence detection, the sections were incubated with the goat anti-mouse CCR9 primary antibody combined with the CD68, CD3, or α-actinin antibodies and secondary antibodies (FITC-conjugated anti-goat and TRITC-conjugated anti-mouse).

### Evans blue and TTC staining

Evans blue and TTC staining was conducted 24 h after acute myocardial infarction to determine the infarct size. The mice were anesthetized and a median sternotomy was performed. Approximately 2 ml of 2.5% Evans blue dye (Sigma-Aldrich, USA) were injected into the jugular vein to delineate the myocardial perfusion region. The heart was then excised, rinsed in saline, arrested in 10% KCl and frozen at −20 °C for 15 min. The frozen ventricle was cut transversely into four slices and stained with 2% 2,3,5-triphenyltetrazolium chloride (TTC, T8877, Sigma-Aldrich, St Louis, MO, USA) for 15 min at 37 °C. The infarct area was expected to be free of staining, and the normal tissue to be stained in blue. The sections were imaged and the total LV area and the infarct area were quantified using the Image-Pro Plus software (Version 6.0).

### Tunel staining

Terminal deoxynucleotidyl transferase-mediated dUTP nick-end labelling (TUNEL) assays were used to examine cardiomyocyte apoptosis, according to the manufacturer’s protocol. The TUNEL-positive cardiomyocytes in peri-infarct area were observed using a fluorescent microscope. Six fields in each heart slide were randomly selected, and the TUNEL-positive cardiomyocytes were counted under high-power magnification (x400).

### Quantitative real-time PCR (qRT-PCR) and western blot analysis

Total LV RNA was purified from the LV samples using TRIzol reagent (15596-018, Invitrogen). Ribonucleic acids (RNAs) were transcribed into complementary DNAs (cDNAs) with the PrimeScript RT reagent Kit (#K1622, Fermentas). Then, qRT-PCR was conducted in a 20 μl reaction system containing cDNA, forward primer, reverse primer and SYBR Green/Fluorescein qPCR Master Mix (#K0242, Fermentas). The relative gene expression was normalized to the internal reference gene β-actin. The sequences of the primers used for RT-PCR in this experiment are as described in Supplementary Table S1.

Total proteins were extracted from the frozen LV tissues. Protein concentrations were determined and normalized using the Bicinchoninic Acid (BCA) Protein Assay Kit (AS1086, ASPEN). Proteins (40 μg) were separated by sodium dodecylsulphate (SDS)-polyacrylamide gel electrophoresis (PAGE), transferred onto a polyvinylidene difluoride (PVDF) membrane, and incubated with primary antibodies (Supplementary Table S2) overnight at 4 °C. Secondary antibodies were incubated with the membranes for 30 min at room temperature. Enhanced chemiluminescence was used to visualize the signals.

### Telemetry

A small telemetric biopotential transmitter (EA-F20, Data Sciences International, St. Paul, MN) was intraperitoneally implanted after the mice were lightly anesthetized. The leads were introduced into the right shoulder and left apex (lead II) through an s.c. tunnel. The recordings began after the mice had recovered from the anesthesia for >24 hours, and electrocardiogram (ECG) recordings were acquired continuously for 24 h. The signals were recorded by the data acquisition system (DSI, US) and analyzed using the P3 software^60^.

### Preparation of Langendorff-perfused hearts, monophasic action potential recordings, and ventricular arrhythmia inducibility

The hearts were removed and rinsed. The aorta was quickly hung on the Langendorff-perfusion system (AD Instruments, Australia) and perfused with Tyrode’s solution (mM: NaCl 130; KCl 5.4; CaCl2 1.8; MgCl2 1; Na2HPO4 0.3; HEPES 10; and glucose 10; pH adjusted to 7.4 with NaOH) at a rate of 2.5 ml/min at 37 °C. The hearts were perfused for 20 min to allow them to recover their regular spontaneous rhythm before the electrophysiology tests. A pair of platinum-stimulating electrodes were positioned on the basal surface of the right ventricle to delivery regular pacing and a custom-made Ag-AgCl electrode consisting of two 0.25-mm, Teflon-coated silver wires was located at LV peri-infarct zone to record the monophasic action potential (MAP). The S1-S1 pacing protocol was conducted at a given pacing cycle length (PCL) with a series of pulse trains. The PCL ranged from 150 ms to 30 ms, with successive 10 ms decreases. The pacing at each PCL lasted for 30 seconds, followed by a 30 seconds resting period to avoid pacing memory. The 90% action potential duration (APD90) at the PCL of 150 ms was defined as the average 90% repolarization time for at least six successive MAPs. The APD alternans (ALT) was determined by 2 consecutive beats whose APD90 differed by at least 5% over 10 beats. The APD alternans threshold was defined as the maximal PCL (PCLmax) that induced an APD alternans. Burst pacing (2 ms pulses at 50 Hz, 2 s burst duration) was used to induce ventricular arrhythmia in infarct border zone of the LV. Ventricular arrhythmia (VA) was defined as continuous ventricular contractions of 2 seconds or more. Nonsustained ventricular arrhythmia was defined as contractions that lasted for 2 to 30 s and sustained ventricular arrhythmia was defined as contractions that lasted for more than 30 s^61^.

### Statistical analysis

The numerical data were expressed as the means ± SEM. Comparisons between two groups were performed using unpaired Student’s t-test with SPSS17.0. Differences among groups were assessed using one-way analysis of variance (ANOVA), followed by Tukey’s post hoc test. Cross Tabulation (Chi-Square Test) with Fisher’s exact test was used for the categorical variables. Statistical significance was defined as P < 0.05.

## Additional Information

**How to cite this article**: Huang, Y. *et al*. Abrogation of CC chemokine receptor 9 ameliorates ventricular remodeling in mice after myocardial infarction. *Sci. Rep.* **6**, 32660; doi: 10.1038/srep32660 (2016).

## Supplementary Material

Supplementary Information

## References

[b1] Thygesen K. . Third universal definition of myocardial infarction. Journal of the American College of Cardiology 60, 1581–1598 (2012). 22958960 10.1016/j.jacc.2012.08.001

[b2] Frangogiannis N. G. The inflammatory response in myocardial injury, repair, and remodelling. Nature Reviews Cardiology 11, 255–265 (2014). 24663091 10.1038/nrcardio.2014.28 PMC4407144

[b3] Buja L. M. & Vander Heide R. S. Pathobiology of cardiovascular diseases: past, present, and future perspectives. Cardiovascular Pathology 25, 214–220 (2016). 26897485 10.1016/j.carpath.2016.01.007

[b4] Thiene G. & Basso C. Myocardial infarction: a paradigm of success in modern medicine. Cardiovascular Pathology 19, 1–5 (2010). 19775916 10.1016/j.carpath.2009.08.002

[b5] Clark-Lewis I. . Structure-activity relationships of chemokines. Journal of leukocyte biology 57, 703–711 (1995). 7759949 10.1002/jlb.57.5.703

[b6] Frangogiannis N. Chemokines in the ischemic myocardium: from inflammation to fibrosis. Inflammation Research 53, 585–595 (2004). 15693606 10.1007/s00011-004-1298-5

[b7] White G. E., Iqbal A. J. & Greaves D. R. CC chemokine receptors and chronic inflammation—therapeutic opportunities and pharmacological challenges. Pharmacological reviews 65, 47–89 (2013). 23300131 10.1124/pr.111.005074

[b8] Schulz O., Hammerschmidt S. I., Moschovakis G. L. & Forster R. Chemokines and Chemokine Receptors in Lymphoid Tissue Dynamics. Annual review of immunology, 10.1146/annurev-immunol-041015-055649 (2016). 26907216

[b9] Zhong Y. . Expression of CC chemokine receptor 9 predicts poor prognosis in patients with lung adenocarcinoma. Diagnostic pathology 10, 101, 10.1186/s13000-015-0341-x (2015). 26168791 PMC4501107

[b10] Zaballos A., Gutierrez J., Varona R., Ardavin C. & Marquez G. Cutting edge: identification of the orphan chemokine receptor GPR-9-6 as CCR9, the receptor for the chemokine TECK. Journal of immunology 162, 5671–5675 (1999). 10229797

[b11] Vicari A. P. . TECK: a novel CC chemokine specifically expressed by thymic dendritic cells and potentially involved in T cell development. Immunity 7, 291–301 (1997). 9285413 10.1016/s1074-7613(00)80531-2

[b12] Carramolino L. . Expression of CCR9 beta-chemokine receptor is modulated in thymocyte differentiation and is selectively maintained in CD8(+) T cells from secondary lymphoid organs. Blood 97, 850–857 (2001). 11159507 10.1182/blood.v97.4.850

[b13] Papadakis K. A. . CCR9-positive lymphocytes and thymus-expressed chemokine distinguish small bowel from colonic Crohn’s disease. Gastroenterology 121, 246–254 (2001). 11487533 10.1053/gast.2001.27154

[b14] Nakamoto N. . CCR9+ macrophages are required for acute liver inflammation in mouse models of hepatitis. Gastroenterology 142, 366–376, 10.1053/j.gastro.2011.10.039 (2012). 22079594

[b15] Schmutz C. . Monocytes/macrophages express chemokine receptor CCR9 in rheumatoid arthritis and CCL25 stimulates their differentiation. Arthritis research & therapy 12, R161, 10.1186/ar3120 (2010). 20738854 PMC2945064

[b16] Li J. . Anti-CCL25 antibody prolongs skin allograft survival by blocking CCR9 expression and impairing splenic T-cell function. Archivum immunologiae et therapiae experimentalis 61, 237–244, 10.1007/s00005-013-0223-4 (2013). 23456208

[b17] Tu Z. . CCR9 in cancer: oncogenic role and therapeutic targeting. Journal of hematology & oncology 9, 10, 10.1186/s13045-016-0236-7 (2016). 26879872 PMC4754913

[b18] Abd Alla J. . Angiotensin-converting enzyme inhibition down-regulates the pro-atherogenic chemokine receptor 9 (CCR9)-chemokine ligand 25 (CCL25) axis. The Journal of biological chemistry 285, 23496–23505, 10.1074/jbc.M110.117481 (2010). 20504763 PMC2906340

[b19] Costa M. F. . CCL25 induces alpha(4)beta(7) integrin-dependent migration of IL-17(+) gammadelta T lymphocytes during an allergic reaction. European journal of immunology 42, 1250–1260, 10.1002/eji.201142021 (2012). 22539297

[b20] McGuire H. M. . A subset of interleukin-21+ chemokine receptor CCR9+ T helper cells target accessory organs of the digestive system in autoimmunity. Immunity 34, 602–615, 10.1016/j.immuni.2011.01.021 (2011). 21511186

[b21] Chen H. J. . Chemokine 25-induced signaling suppresses colon cancer invasion and metastasis. The Journal of clinical investigation 122, 3184–3196, 10.1172/JCI62110 (2012). 22863617 PMC3428084

[b22] Apostolaki M. . Role of beta7 integrin and the chemokine/chemokine receptor pair CCL25/CCR9 in modeled TNF-dependent Crohn’s disease. Gastroenterology 134, 2025–2035, 10.1053/j.gastro.2008.02.085 (2008). 18439426

[b23] Yokoyama W. . Abrogation of CC chemokine receptor 9 ameliorates collagen-induced arthritis of mice. Arthritis research & therapy 16, 445, 10.1186/s13075-014-0445-9 (2014). 25248373 PMC4201712

[b24] Wurbel M. A., McIntire M. G., Dwyer P. & Fiebiger E. CCL25/CCR9 interactions regulate large intestinal inflammation in a murine model of acute colitis. PloS one 6, e16442, 10.1371/journal.pone.0016442 (2011). 21283540 PMC3026821

[b25] Frangogiannis N. G. & Entman M. L. Chemokines in myocardial ischemia. Trends in cardiovascular medicine 15, 163–169, 10.1016/j.tcm.2005.06.005 (2005). 16165012

[b26] Farouk S. S., Rader D. J., Reilly M. P. & Mehta N. N. CXCL12: a new player in coronary disease identified through human genetics. Trends in cardiovascular medicine 20, 204–209, 10.1016/j.tcm.2011.08.002 (2010). 22137643 PMC3235407

[b27] Altara R. . Emerging importance of chemokine receptor CXCR3 and its ligands in cardiovascular diseases. Clinical science 130, 463–478, 10.1042/CS20150666 (2016). 26888559

[b28] Liehn E. A. . A new monocyte chemotactic protein-1/chemokine CC motif ligand-2 competitor limiting neointima formation and myocardial ischemia/reperfusion injury in mice. Journal of the American College of Cardiology 56, 1847–1857, 10.1016/j.jacc.2010.04.066 (2010). 21087715

[b29] Montecucco F. . CC chemokine CCL5 plays a central role impacting infarct size and post-infarction heart failure in mice. European heart journal 33, 1964–1974, 10.1093/eurheartj/ehr127 (2012). 21606075

[b30] Xuan W. . Detrimental effect of fractalkine on myocardial ischaemia and heart failure. Cardiovascular research 92, 385–393, 10.1093/cvr/cvr221 (2011). 21840883

[b31] Boyle E. M. Jr. . Inhibition of interleukin-8 blocks myocardial ischemia-reperfusion injury. The Journal of thoracic and cardiovascular surgery 116, 114–121, 10.1016/S0022-5223(98)70249-1 (1998). 9671905

[b32] Kaikita K. . Targeted deletion of CC chemokine receptor 2 attenuates left ventricular remodeling after experimental myocardial infarction. The American journal of pathology 165, 439–447, 10.1016/S0002-9440(10)63309-3 (2004). 15277218 PMC1618584

[b33] Dobaczewski M., Xia Y., Bujak M., Gonzalez-Quesada C. & Frangogiannis N. G. CCR5 signaling suppresses inflammation and reduces adverse remodeling of the infarcted heart, mediating recruitment of regulatory T cells. The American journal of pathology 176, 2177–2187, 10.2353/ajpath.2010.090759 (2010). 20382703 PMC2861083

[b34] Norment A. M., Bogatzki L. Y., Gantner B. N. & Bevan M. J. Murine CCR9, a chemokine receptor for thymus-expressed chemokine that is up-regulated following pre-TCR signaling. Journal of immunology 164, 639–648 (2000). 10.4049/jimmunol.164.2.639 10623805

[b35] Liu J. X., Cao X., Tang Y. C., Liu Y. & Tang F. R. CCR7, CCR8, CCR9 and CCR10 in the mouse hippocampal CA1 area and the dentate gyrus during and after pilocarpine-induced status epilepticus. Journal of neurochemistry 100, 1072–1088, 10.1111/j.1471-4159.2006.04272.x (2007). 17181556

[b36] Xu Z., Mei F., Liu H., Sun C. & Zheng Z. C-C Motif Chemokine Receptor 9 Exacerbates Pressure Overload-Induced Cardiac Hypertrophy and Dysfunction. Journal of the American Heart Association 5, 10.1161/JAHA.116.003342 (2016). PMC4889199 27146447

[b37] Saxena A., Russo I. & Frangogiannis N. G. Inflammation as a therapeutic target in myocardial infarction: learning from past failures to meet future challenges. Translational research : the journal of laboratory and clinical medicine 167, 152–166, 10.1016/j.trsl.2015.07.002 (2016). 26241027 PMC4684426

[b38] Hinz M. & Scheidereit C. The IkappaB kinase complex in NF-kappaB regulation and beyond. EMBO reports 15, 46–61, 10.1002/embr.201337983 (2014). 24375677 PMC4303448

[b39] Hamid T. . Cardiomyocyte NF-kappaB p65 promotes adverse remodelling, apoptosis, and endoplasmic reticulum stress in heart failure. Cardiovascular research 89, 129–138, 10.1093/cvr/cvq274 (2011). 20797985 PMC3002872

[b40] Ghosh S. & Hayden M. S. New regulators of NF-kappaB in inflammation. Nature reviews. Immunology 8, 837–848, 10.1038/nri2423 (2008). 18927578

[b41] Onai Y. . Inhibition of NF-{kappa}B improves left ventricular remodeling and cardiac dysfunction after myocardial infarction. American journal of physiology. Heart and circulatory physiology 292, H530–538, 10.1152/ajpheart.00549.2006 (2007). 16920808

[b42] Sun S. J., Wu X. P., Song H. L. & Li G. Q. Baicalin ameliorates isoproterenol-induced acute myocardial infarction through iNOS, inflammation, oxidative stress and P38MAPK pathway in rat. International journal of clinical and experimental medicine 8, 22063–22072 (2015). 26885181 PMC4729967

[b43] Hunter J. J., Tanaka N., Rockman H. A., Ross J. Jr. & Chien K. R. Ventricular expression of a MLC-2v-ras fusion gene induces cardiac hypertrophy and selective diastolic dysfunction in transgenic mice. The Journal of biological chemistry 270, 23173–23178 (1995). 7559464 10.1074/jbc.270.39.23173

[b44] Cowan K. J. & Storey K. B. Mitogen-activated protein kinases: new signaling pathways functioning in cellular responses to environmental stress. The Journal of experimental biology 206, 1107–1115 (2003). 12604570 10.1242/jeb.00220

[b45] Kyriakis J. M. & Avruch J. Mammalian MAPK signal transduction pathways activated by stress and inflammation: a 10-year update. Physiological reviews 92, 689–737, 10.1152/physrev.00028.2011 (2012). 22535895

[b46] Yao Y. Y., Yin H., Shen B., Chao L. & Chao J. Tissue kallikrein infusion prevents cardiomyocyte apoptosis, inflammation and ventricular remodeling after myocardial infarction. Regulatory peptides 140, 12–20, 10.1016/j.regpep.2006.11.020 (2007). 17196272 PMC1876786

[b47] Rose B. A., Force T. & Wang Y. Mitogen-activated protein kinase signaling in the heart: angels versus demons in a heart-breaking tale. Physiological reviews 90, 1507–1546, 10.1152/physrev.00054.2009 (2010). 20959622 PMC3808831

[b48] Yue T. L. . Inhibition of extracellular signal-regulated kinase enhances Ischemia/Reoxygenation-induced apoptosis in cultured cardiac myocytes and exaggerates reperfusion injury in isolated perfused heart. Circulation research 86, 692–699 (2000). 10747006 10.1161/01.res.86.6.692

[b49] Pan Z. . Scutellarin alleviates interstitial fibrosis and cardiac dysfunction of infarct rats by inhibiting TGFbeta1 expression and activation of p38-MAPK and ERK1/2. British journal of pharmacology 162, 688–700, 10.1111/j.1476-5381.2010.01070.x (2011). 20942814 PMC3041257

[b50] Marber M. S., Rose B. & Wang Y. The p38 mitogen-activated protein kinase pathway–a potential target for intervention in infarction, hypertrophy, and heart failure. Journal of molecular and cellular cardiology 51, 485–490, 10.1016/j.yjmcc.2010.10.021 (2011). 21062627 PMC3061241

[b51] Tien Y. C. . Carthamus tinctorius L. prevents LPS-induced TNFalpha signaling activation and cell apoptosis through JNK1/2-NFkappaB pathway inhibition in H9c2 cardiomyoblast cells. Journal of ethnopharmacology 130, 505–513, 10.1016/j.jep.2010.05.038 (2010). 20538053

[b52] Francis Stuart S. D., De Jesus N. M., Lindsey M. L. & Ripplinger C. M. The crossroads of inflammation, fibrosis, and arrhythmia following myocardial infarction. Journal of molecular and cellular cardiology 91, 114–122, 10.1016/j.yjmcc.2015.12.024 (2016). 26739214 PMC4764395

[b53] Pinto J. M. & Boyden P. A. Electrical remodeling in ischemia and infarction. Cardiovascular research 42, 284–297 (1999). 10533567 10.1016/s0008-6363(99)00013-9

[b54] Peters N. S., Coromilas J., Severs N. J. & Wit A. L. Disturbed connexin43 gap junction distribution correlates with the location of reentrant circuits in the epicardial border zone of healing canine infarcts that cause ventricular tachycardia. Circulation 95, 988–996 (1997). 9054762 10.1161/01.cir.95.4.988

[b55] Streitner F. . Prospective study of interleukin-6 and the risk of malignant ventricular tachyarrhythmia in ICD-recipients–a pilot study. Cytokine 40, 30–34, 10.1016/j.cyto.2007.07.187 (2007). 17851087

[b56] Streitner F. . Role of proinflammatory markers and NT-proBNP in patients with an implantable cardioverter-defibrillator and an electrical storm. Cytokine 47, 166–172, 10.1016/j.cyto.2009.06.003 (2009). 19604708

[b57] Maradit-Kremers H. . Increased unrecognized coronary heart disease and sudden deaths in rheumatoid arthritis: a population-based cohort study. Arthritis and rheumatism 52, 402–411, 10.1002/art.20853 (2005). 15693010

[b58] Li L., Weng Z., Yao C., Song Y. & Ma T. Aquaporin-1 Deficiency Protects Against Myocardial Infarction by Reducing Both Edema and Apoptosis in Mice. Scientific reports 5, 13807, 10.1038/srep13807 (2015). 26348407 PMC4562302

[b59] Bao M. W. . Cardioprotective role of growth/differentiation factor 1 in post-infarction left ventricular remodelling and dysfunction. The Journal of pathology 236, 360–372, 10.1002/path.4523 (2015). 25726944

[b60] Ying S. . Alk7 Depleted Mice Exhibit Prolonged Cardiac Repolarization and Are Predisposed to Ventricular Arrhythmia. PloS one 11, e0149205, 10.1371/journal.pone.0149205 (2016). 26882027 PMC4755580

[b61] Wang D. . Chronic Administration of Catestatin Improves Autonomic Function and Exerts Cardioprotective Effects in Myocardial Infarction Rats. Journal of cardiovascular pharmacology and therapeutics, 10.1177/1074248416628676 (2016). 26821570

