# Supplementary material for: Abrogation of CC chemokine receptor 9 ameliorates ventricular remodeling in mice after myocardial infarction
Source: Sci Rep. 2016 Sep 2;6:32660. doi: 10.1038/srep32660 (PMC5009347; doi:10.1038/srep32660)
Supplement: Supplementary Information [file srep32660-s1.pdf]

Abrogation of CC chemokine receptor 9 ameliorates ventricular remodeling in mice after myocardial infarction

Yan Huang[1,2,3], Dandan Wang[1,2,3], Xin Wang[1], Yijie Zhang[1,2,3], Tao Liu[1,2,3], Yuting Chen[2,3], Yanhong Tang[1,2,3], Teng Wang[1,2,3], Dan Hu[2,3,4], Congxin Huang[1,2,3]*

[1]Department of Cardiology, Renmin Hospital of Wuhan University, Wuhan 430060, PR China;

[2]Cardiovascular Research Institute, Wuhan University, Wuhan 430060, PR China;

[3]Hubei Key Laboratory of Cardiology, Wuhan 430060, PR China;

[4]Masonic Medical Research Laboratory, Utica, NY, USA.

Correspondence to: Congxin Huang, MD, PhD, Professor and Director, Department of Cardiology, Renmin Hospital of Wuhan University; Cardiovascular Research Institute, Wuhan University; Hubei Key Laboratory of Cardiology, Jiefang Road 238, Wuhan 430060, PR China. Telephone: (+86)13907131546. E-mail: huangcongxin@vip.163.com

Supplementary Table S1: List of primers used for qRT-PCR

Primer name(mouse)	Forward Primer (5'-3')	Reverse Primer (5'-3')
β-actin	CACGATGGAGGGGCCGGACTCATC	TAAAGACCTCTATGCCAACACAGT
CCL25	GAATGTGAAGAGGGCGATGA	CTCACGCTTGTACTGTTGGG
CCR9	TGGCTTGTGTTCATTGTGGG	CAGAAGGGAAGAGTGGCAAG
IL-1β	GCCCATCCTCTGTGACTCAT	AGGCCACAGGTATTTTGTCG
IL-6	GTTGCCTTCTTGGGACTGATG	GTATAGACAGGTCTGTTGGGAG
TNF-*a*	CGTCAGCCGATTTGCTATCT	CGGACTCCGCAAAGTCTAAG
ANP	ACCTGCTAGACCACCTGGAG	CCTTGGCTGTTATCTTCGGTACCGG
BNP	GAGGTCACTCCTATCCTCTGG	GCCATTTCCTCCGACTTTTCTC
β-MHC	CCGAGTCCCAGGTCAACAA	CTTCACGGGCACCCTTGGA
CTGF	TGACCCCTGCGACCCACA	TACACCGACCCACCGAAGACACAG
Collagen I	AGGCTTCAGTGGTTTGGATG	CACCAACAGCACCATCGTTA
Collagen III	CCCAACCCAGAGATCCCATT	GAAGCACAGGAGCAGGTGTAGA

Supplementary Table S2: Antibodies and conditions used for Western Blot Analysis

Protein	kDA	Supplier	Cat. Number	Source	Clonality	Dilution
CCR9	43	Abcam	ab1662	Goat	polyclonal	1:1000
CCL25	17	Santa Cruz	Sc-80344	Rat	monoclonal	1:200
Bax	20	Cell Signaling Technology	#2772	Rabbit	polyclonal	1:1000
Bcl2	26	Cell Signaling Technology	#2870	Rabbit	monoclonal	1:1000
Capase3	37	Cell Signaling Technology	#9662	Rabbit	polyclonal	1:500
C-Casp3	19/17	Cell Signaling Technology	#9664	Rabbit	monoclonal	1:1000
T-IκBa	40	ABclonal	A1187	Rabbit	polyclonal	1:1000
P-IκBa	40	Abcam	ab133462	Rabbit	monoclonal	1:1000
T-p65	65	Cell Signaling Technology	#6956	Mouse	monoclonal	1:1000
P-p65	65	Abcam	ab131109	Rabbit	polyclonal	1:1000
T-p38	42	Abcam	ab170099	Rabbit	monoclonal	1:1000
P-p38	42	Cell Signaling Technology	#4511	Rabbit	monoclonal	1:500
T-JNK1/2	54/46	Abcam	ab37228	Mouse	monoclonal	1:1000
P-JNK1/2	54/46	Abcam	ab131499	Rabbit	polyclonal	1:1000
T-ERK1/2	44/42	Cell Signaling Technology	#4695	Rabbit	monoclonal	1:1000
P-ERK1/2	44/42	Cell Signaling Technology	#4370	Rabbit	monoclonal	1:1000
GAPDH	37	Abcam	37168	Rabbit	monoclonal	1:10000

Supplementary Table S3: PCR protocol for CCR9-KO identification

Primer1: Ccr9-Common: ACAGCTGTAGAAGTTCATCTTGTAC			
Primer2: Ccr9-WT: GGGCACCTTGGGCAACAGCCTGGTC			
Primer3: Ccr9-KO: GGGTGGGATTAGATAAATGCCTGCTCT			
Primer set		Product size(bp)	Product
Primer1	Primer2	217	WT
Primer1	Primer3	380	KO

Supplementary Figure S1

Marker -/- -/+ +/+ Marker

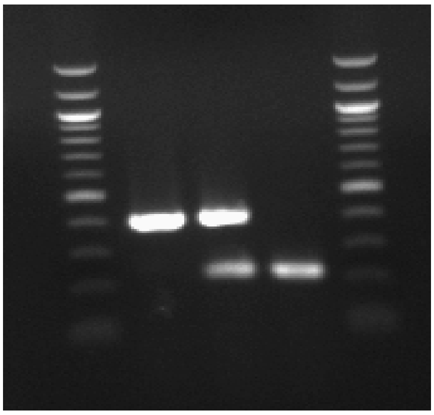

KO: 380 bp

WT: 217 bp

Supplementary Figure S2:

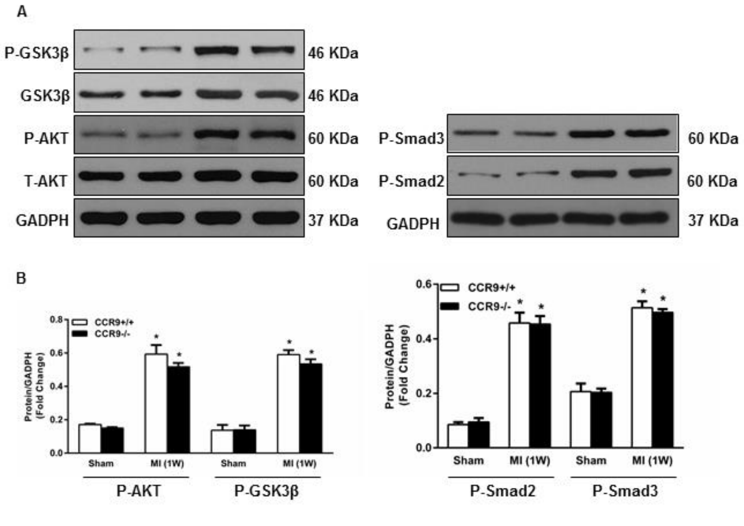

Supplementary Figure S3:

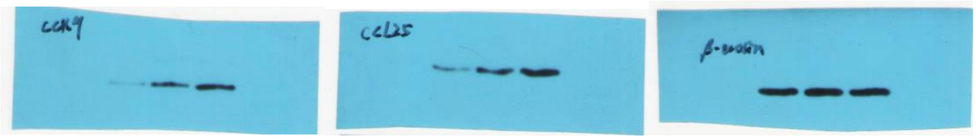

Supplementary Figure S4:

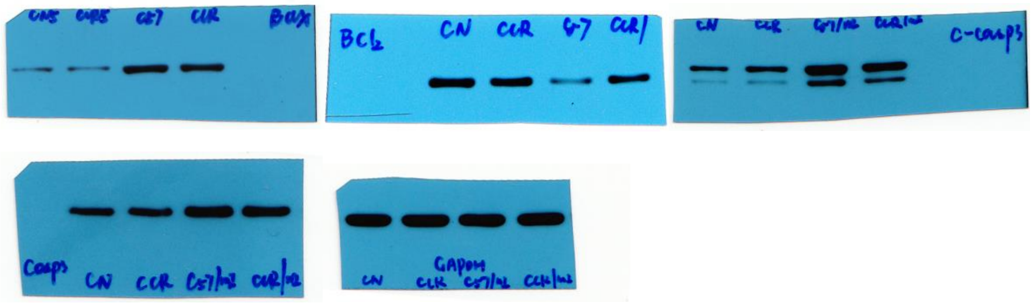

Supplementary Figure S5:

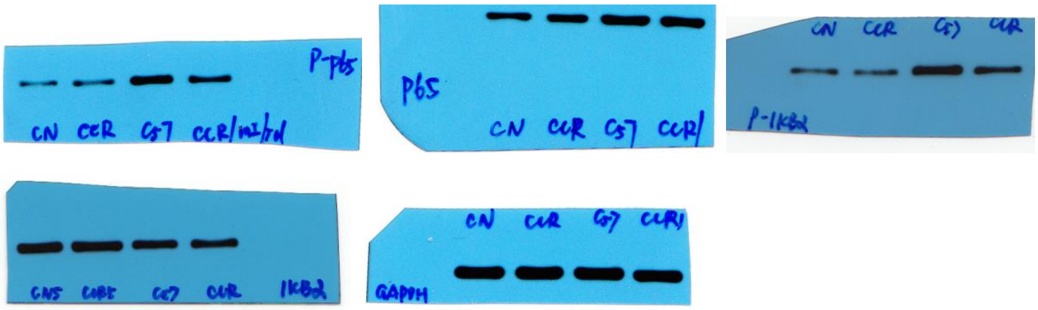

Supplementary Figure S6:

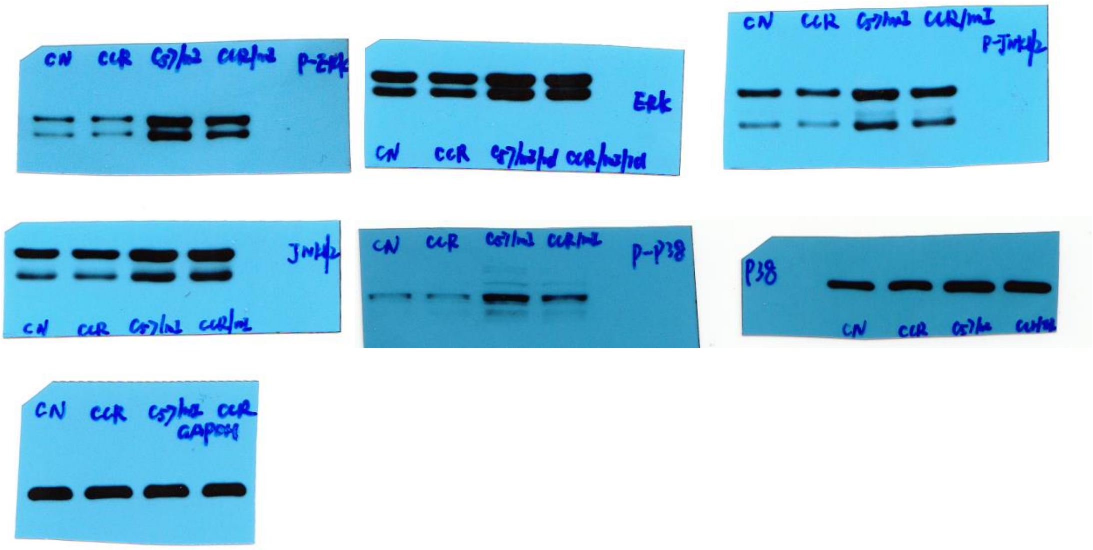

Figure legends

Figure S1: DNA-PCR analysis for identification of CCR9+/+, CCR9+/- and CCR9-/- mice using the pair of primers shown in Supplementary Table S3. The amplified PCR product size is 380bp for CCR9-/- mice, 217bp for CCR9+/+ mice.

Figure S2: The loss of CCR9 didn't change expression levels of proteins involved in AKT and Smad signaling pathways. A) Representative western blots of AKT, GSK3βand Smad2, Smad3 in CCR9+/+ and CCR9-/- mouse heart tissues 1 week after the sham operation or MI surgery. B) Statistical analysis of P-AKT, P-GSK3β, P-Smad2 and P-Smad3 in CCR9+/+ and CCR9-/- mouse heart tissues 1 week after the sham operation or MI surgery. (n=4, *P < 0.05 vs. their littermate shams).

Figure S3: The original bands for Figure 1C in the main manuscript.

Figure S4: The original bands for Figure 3B in the main manuscript.

Figure S5: The original bands for Figure 4D in the main manuscript.

Figure S6: The original bands for Figure 6 in the main manuscript.
